# Dynamic Analysis of a Uniform Microbeam Resting on a Nonlinear Foundation Considering Its Curvature Subjected to a Mechanical Impact and Electromagnetic Actuation

**DOI:** 10.3390/mi15080969

**Published:** 2024-07-29

**Authors:** Nicolae Herisanu, Bogdan Marinca, Vasile Marinca

**Affiliations:** 1 Department of Mechanics and Strength of Materials, University Politehnica Timisoara, 300222 Timișoara, Romania; vasile.marinca@upt.ro; 2 Center for Advanced and Fundamental Technical Research, Romanian Academy, 300222 Timişoara, Romania; 3 Department of Applied Electronics, University Politehnica Timisoara, 300006 Timișoara, Romania; bogdan.marinca@upt.ro

**Keywords:** mechanical impact, electromagnetic microactuator, Winkler–Pasternak foundation, local stability

## Abstract

This study proposes an investigation into the nonlinear vibration of a simply supported, flexible, uniform microbeam associated with its curvature considering the mechanical impact, the electromagnetic actuation, the nonlinear Winkler–Pasternak foundation, and the longitudinal magnetic field. The governing differential equations and the boundary conditions are modeled within the framework of a Euler–Bernoulli beam considering an element of the length of the beam at rest and using the second-order approximation of the deflected beam and the Galerkin–Bubnov procedure. In this work, we present a novel characterization of the microbeam and a novel method to solve the nonlinear vibration of the microactuator. The resulting equation of this complex problem is studied using the Optimal Homotopy Asymptotic Method, employing some auxiliary functions derived from the terms that appear in the equation of motion. An explicit closed-form analytical solution is proposed, proving that our procedure is a powerful tool for solving a nonlinear problem without the presence of small or large parameters. The presence of some convergence-control parameters assures the rapid convergence of the solutions. These parameters are evaluated using some rigorous mathematical procedures. The present approach is very accurate and easy to implement, even for complicated nonlinear problems. The local stability near the primary resonance is studied.

## 1. Introduction

The flexural vibration of a microbeam under mechanical impact, electromagnetic actuation, a moving body, or a nonlinear foundation is interesting for basic research on vibration problems that has in view their practical interest. The main component of an electromagnetic actuator is the solenoid actuator. This is comprised of housing, a moving rod, coils, and a spring [1]. The moving rod is made of a ferroelectric material that is surrounded by coils. When a voltage is applied through the coils, a current flow appears through the coils, which results in an electromagnetic force exerted on the moving rod, causing the rod to move. An inherent nonlinear relationship between displacement and current input exists for solenoids.

Some previous studies have been the works of Abiala [2], which used the finite element method and Newmark’s integration to obtain the dynamic response of a beam under uniformly distributed moving loads. The effect of electromagnetic actuation on the dynamics of a single-sided Hertzian contact-forced oscillator is explored near primary resonance and secondary resonance by Bichri et al. [3].

Muscia [4] obtained an engineering solution to manufacture electromagnetic linear actuators for moving rudders and fin stabilizers on military ships. The finite element method analysis helped them to find the most convenient solution for joining the pole pieces to the respective bars. Bichri et al. [5] considered the effects of combined DC and fast AC electromagnetic actuations on the dynamic behavior of an excited cantilever beam considering a single-mode approximation. Also, the influence of the fast AC actuation and the air gap on the nonlinear behavior of the system was analyzed. The time-stepping solution of the electromagnetic field model resulting in the magnetic force densities, computed from the Lorentz force and based on the space–time variation of the induced eddy current, was employed by Abba and Rachek [6]. In this way, this framework was dedicated to predicting the wave magnetic force density for the mechanical deformations occurring in electromagnetic actuators.

A novel nonlinear lumped parameter model of the electromagnetic actuator is proposed by Zuo et al. [7] for the engine’s idling or low-speed condition. The LuGre friction model is applied to describe the friction during the mover’s reciprocating motion. A specific experimental scheme is proposed for validation in which a suspended ring-shaped iron is added as the actuator’s load. Another novel electromagnetic actuator that has been designed to produce energy via vibrations while suppressing said vibrations, which can be installed in many kinds of mechanisms to improve machining accuracy and reduce energy loss, is employed by Wei et al. [8]. Based on this prototype, the characteristics of the electromagnetic actuator are employed by means of measuring experiments. On the other hand, a novel, bistable, nonlinear electromagnetic actuator with an elastic boundary to enhance the actuation performance when controlled by a harmonic input signal is attempted by Zhang et al. [9]. This model has an inclined spring, one end of which is supported by an elastic boundary, which produces bistable nonlinearity to realize the large-amplitude inter-well actuation responses. The governing equations are controlled by a harmonic input signal.

A self-sensing electromagnetic actuator for vibration control of flexible structures is studied by Verma et al. [10]. This scheme does not require any modifications in the design of the electromagnetic actuator and has the advantage that the voltage drop across the coil can be measured in relation to motion. Seebacher et al. [11] introduced a pseudo-density topology optimization method in nonlinear electromagnetism that follows the Solid Isotropic Material with Penalization procedure. The adjoint technique is used to determine the sensitivity of the objective functions. An applied linear characteristic of the ferromagnetic material results in a small or wrongly actuated electromagnetic dimension. The mathematical model of lateral vibration of a cantilever pipe discharging fluid using an electromagnetic actuator of the transformer type in the form of a partial differential equation coupled with two ordinary differential equations is presented by Szmidt et al. [12]. The system is discretized in a set of ordinary differential equations and is solved numerically. The critical velocity and the amplitude of post-critical vibrations are determined and validated experimentally.

Al Bakri et al. [13] considered a sliding mode control method to design the closed-loop coil voltage such that the nonlinear system of an electromagnetic actuator slides along a certain sliding surface. The reliability of the system was approved under a wide range of steady-state actuator position dispersions and coil voltage disturbances using the Monte Carlo simulation method.

A novel miniaturized and modular dual-axis electromagnetic actuator for several applications is proposed by Mansour et al. [14]. The modularity of this actuator gives an extra privilege to connect several modules together, providing more potential applications, and can be used to generate a quadrupled robot. The robot shows a high level of manipulability and has a light weight and a small size. A new concept of two-way electromagnetic actuation with the help of magnetic damping, which can extend its arm on both sides to facilitate active suspension mechanisms in both humps and potholes, has been derived by Prajwal et al. [15]. Catia V5 software was used to design and simulate the actuator model. Repinaldo et al. [16] investigated the parameter identification and application of the active vibration control technique within a structure with two degrees of freedom using an electromagnetic actuator. The neuro-fuzzy control theory was applied to reduce the amplitude of vibration in the structure when subjected to an impulsive force.

Konig et al. [17] explored the design of a hysteresis-compensated self-sensing algorithm with low computational effort and integrator-based direct inductance. The measurement technique was applied for the resource-efficient estimation of the incremental inductance. Also, a modified Prandtl–Ishlinski approach was used for modeling this hysteretic behavior. Yang et al. [18] examined the broadband active vibration of a bistable, nonlinear electromagnetic actuator with an elastic boundary. A new feedback control law was proposed to control the actuator’s input curves to significantly attenuate the broadband vibration transmissibility from the base excitation to the actuator’s movements. The input-to-state stability of the control law for any non-negative control weights was proven.

Zhang et al. [19] proposed the physical model of the actuator via Ampere’s loop law, the continuity principle of magnetic flux, and Ohm’s law of magnetic circuits. To enhance hysteresis model accuracy, the magnetic flux leakage coefficients of the actuator with different positions were obtained. The experimental results demonstrate that the trajectory and tracking errors compared with the feedback control and the unoptimized hysteresis method feedforward control of the optimized hysteresis were substantially reduced. Lim et al. [20] investigated the stochastic vibration responses of a bistable electromagnetic actuator with an elastic boundary controlled by Gaussian white noise and low-pass-filtered stochastic noise. This actuator used an oblique spring and another linear spring to realize the bistability and the elastic boundary, respectively.

Jorshari et al. [21] investigated the vibration of single-walled boron nitride nanotube induced by a moving nanoparticle based on the Rayleigh beam model and used the Pasternak substrate to model the elastic medium. Baroudi et al. [22] predicted quantitatively the motion of a soft-beam clamped Euler’s Elastica by adapting the Hencky technique in a regime of large displacement and deformations. A nonlinear planar beam with stretch and shear deformation is described by Ren et al. [23]. The static equations of equilibria are discretized by the finite difference method. The nonlinear vibration of an elastic string with large amplitude and large curvature has been investigated by Zhao et al. [24]. The modified compeer novel form method and the finite difference scheme are used to calculate the critical parameters, the time history diagram, configuration, total length, or fundamental frequency.

The present study is devoted to the nonlinear forced vibration of simply supported, flexible, uniform microbeams. The nonlinearities of the equations are caused by the curvature of the beam, mechanical impact, the electromagnetic actuator, the nonlinear Winkler–Pasternak elastic foundation, and the longitudinal magnetic field. The governing nonlinear differential equations and the corresponding boundary conditions are established for a Euler–Bernoulli beam considering an element of the length of the beam at rest and applying the second-order approximation of the deflected beam and the Galerkin–Bubnov procedure. The two differential equations are reduced to only one nonlinear differential equation, and this equation is solved by means of the Optimal Homotopy Asymptotic Method (OHAM) with the help of so-called auxiliary functions obtained from the terms that appear in the governing equation. A very accurate solution is obtained using a moderate number of convergence-control parameters.

Our original approach is a very powerful tool for solving a nonlinear problem without the presence of any small parameters in the governing equation or in the boundary conditions. The presence of some convergence-control parameters assures the rapid convergence of the approximate solutions after the first iteration. The convergence-control parameters are evaluated using some rigorous mathematical procedures. In general, we have great freedom to select both auxiliary functions and the number of convergence-control parameters. This approach has proven to be very accurate, simple, and easy to implement for any complicated nonlinear problems. The local stability near the primary resonance is studied using the Homotopy Perturbation Method, appealing to the Jacobian and eigenvalues of the system.

## 2. Formulation of the Problem

**Figure 1:**
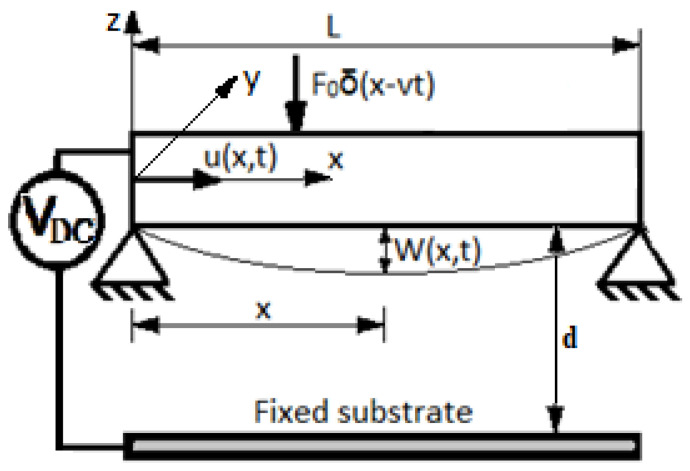
Schematic diagram of the microbeam and electromagnetic microactuator.

In Figure 1 is represented the physical model of a simply supported microbeam of length *L* subjected to a mechanical impact by the transversal constant force, an electromagnetic load *V_DC_*, the nonlinear Winkler–Pasternak elastic foundation *F_WP_*, and longitudinal magnetic field *F_m_*.

The transversal and longitudinal displacements are *W*(*x*,*t*) and *u*(*x*,*t*)*,* respectively. The Young’s modulus *E*, the mass density *ρ*, and the cross-sectional area *A* of the microbeam are supposed to be constant.

Considering an element of length *dx* of the microbeam at rest, the corresponding deflection can be written as

(1) $$ds={\left[{\left(1+\frac{\partial u}{\partial x}\right)}^{2}+{\left(\frac{\partial W}{\partial x}\right)}^{2}\right]}^{1/2}dx$$

The unit vector parallel to the deflected element is of the form

(2) $$\bar{k}=\;\left[\left(1+\frac{\partial u}{\partial x}\right)\bar{i}+\frac{\partial W}{\partial x}\bar{j}\right]\frac{dx}{ds}$$

The length of the microbeam is variable during the motion, such that the tension becomes

(3) $$T=EA\frac{ds-dx}{dx}$$

The governing equations are

(4) $$\rho A\frac{\partial^{2}u}{\partial t^{2}}=-\frac{\partial }{\partial x}(T\bar{k})\bar{i}$$

(5) $$\rho A\frac{\partial^{2}W}{\partial t^{2}}=-\frac{\partial }{\partial x}(T\bar{k})\bar{j}+EI\frac{\partial^{2}}{\partial t^{2}}\left({\frac{\partial^{2}W}{\partial x^{2}}/\sqrt{1+{\left(\frac{\partial W}{\partial x}\right)}^{2}}}^{3}\right)+F_{mi}+F_{em}+F_{WP}+F_{m}$$

where *I* is the moment of inertia of the microbeam cross-section, *F_mi_* is the impact force, and *F_em_* is the electromagnetic actuation.

Neglecting the terms of the forms ${\left(\frac{\partial u}{\partial x}\right)}^{2}$, $\frac{\partial u}{\partial x}\frac{\partial^{2}W}{\partial x^{2}}$, $\frac{\partial^{3}u}{\partial x^{3}}$, $\frac{\partial^{2}u}{\partial x^{2}}$, $\frac{\partial^{2}u}{\partial x^{2}}\frac{\partial^{2}W}{\partial x^{2}}$, and $\frac{\partial^{4}u}{\partial x^{4}}$, from Equation (1), one can obtain

(6) $$\frac{dx}{ds}={\left[{\left(1+u_{x}\right)}^{2}+W_{x}^{2}\right]}^{-1/2}\approx 1-u_{x}-\frac{1}{2}W_{x}^{2}+\frac{3}{8}W_{x}^{4}$$

When

(7) $$e=\frac{ds-dx}{ds}=1-\frac{dx}{ds},$$

then, from Equations (6) and (7), it follows that

(8) $$e=\frac{\partial u}{\partial x}+\frac{1}{2}{\left(\frac{\partial W}{\partial x}\right)}^{2}-\frac{3}{8}{\left(\frac{\partial^{4}W}{\partial x^{4}}\right)}^{4}$$

Equation (4) can be rewritten, taking into account Equations (1)–(3) and (7), as

(9) $$\rho \frac{\partial^{2}u}{\partial x^{2}}+E\frac{\partial e}{\partial x}=0$$

Usually, the longitudinal inertial term $\frac{\partial^{2}u}{\partial x^{2}}$ can be neglected, such that, from Equation (9), it is clear that e is a constant, *e = C*, and, therefore, from Equation (8), it holds that

(10) $$\frac{\partial u}{\partial x}=C-\frac{1}{2}{\left(\frac{\partial W}{\partial x}\right)}^{2}+\frac{3}{8}{\left(\frac{\partial W}{\partial x}\right)}^{4}$$

By integrating the last equation with the boundary conditions

(11) u(0,t)=u(L,t)=0

the constant *C* is given by

(12) $$C=e=\frac{1}{2L}{\int_{0}^{L}\left(\frac{\partial W}{\partial x}\right)}^{2}dx-\frac{3}{8L}{\int_{0}^{L}\left(\frac{\partial W}{\partial x}\right)}^{4}dx$$

Substituting Equation (12) into Equation (5), we obtain

(13) $$\rho A\frac{\partial^{2}W}{\partial t^{2}}+EI\frac{\partial^{2}}{\partial t^{2}}\left({\frac{\partial^{2}W}{\partial x^{2}}/\sqrt{1+{\left(\frac{\partial W}{\partial x}\right)}^{2}}}^{3}\right)=\frac{EA}{2L}\frac{\partial^{2}W}{\partial t^{2}}\left[\int_{0}^{L}{\left(\frac{\partial W}{\partial x}\right)}^{2}dx-\frac{3}{4}\right.\left.\int_{0}^{L}{\left(\frac{\partial W}{\partial x}\right)}^{4}dx\right]+F_{mi}+F_{em}+F_{WP}+F_{m}$$

The term *F_mi_* represents the mechanical impact of load *F*_0_:

(14) $$F_{mi}=F_{0}\delta (x-vt)$$

where *v* is the speed of the load, and *δ* is the Dirac function. The vibration of the microbeam depends on the action of the DC voltage source, such that electromagnetic actuation can be considered as

(15) $$F_{em}=\frac{1}{2}\frac{C_{0}V_{DC}^{2}}{{\left(d-W(x,t)\right)}^{2}}-\frac{1}{2}\frac{C_{0}V_{DC}^{2}}{{\left(d+W(x,t)\right)}^{2}}$$

in which *C*_0_ is the capacitance of the actuator, *d* is the gap width, and *V_DC_* is the voltage.

Expression (15) can be simplified in the following form:

(16) $$\begin{array}{l} \frac{1}{2}\frac{C_{0}V_{DC}^{2}}{{\left(d-W\right)}^{2}}-\frac{1}{2}\frac{C_{0}V_{DC}^{2}}{{\left(d+W\right)}^{2}}=\frac{C_{0}V_{DC}^{2}}{2d^{2}}\left[1/{\left(1-\frac{W}{d}\right)}^{2}-1/{\left(1+\frac{W}{d}\right)}^{2}\right]\approx \\ \approx \frac{2C_{0}V_{DC}^{2}}{d^{2}}\left[\frac{W}{d}+2{\left(\frac{W}{d}\right)}^{3}+3.0125{\left(\frac{W}{d}\right)}^{5}\right] \end{array}$$

The nonlinear elastic Winkler–Pasternak and shear foundation of the beam is represented by

(17) $$F_{WP}=K_{1}W+K_{3}W^{3}-K_{s}\frac{\partial^{2}W}{\partial x^{2}}$$

where *K*_1_ and *K*_3_ are linear and nonlinear coefficients, respectively, and *K_s_* is the shear coefficient of the elastic foundation.

The longitudinal magnetic field is given by magnetic permeability, area *A*, and the component *H_x_* in the x-direction.

(18) $$F_{m}=-\eta AH_{x}^{2}\frac{\partial^{2}W}{\partial x^{2}}$$

According to the governing Equation (13), the term that defines the curvature of the microbeam can be rewritten in the following form:

(19) $${\frac{\partial^{2}W}{\partial x^{2}}/\sqrt{1+{\left(\frac{\partial W}{\partial x}\right)}^{2}}}^{3}\approx \frac{\partial^{2}W}{\partial x^{2}}\left[1-\frac{3}{2}{\left(\frac{\partial W}{\partial x}\right)}^{2}+\frac{15}{8}{\left(\frac{\partial W}{\partial x}\right)}^{4}\right]$$

Inserting Equations (14)–(19) into Equation (13), one retrieves

(20) $$\begin{array}{l} \rho A\frac{\partial^{2}W}{\partial t^{2}}+EI\left[\frac{\partial^{4}W}{\partial x^{4}}\right.-\frac{3}{2}\frac{\partial^{4}W}{\partial x^{4}}{\left(\frac{\partial W}{\partial x}\right)}^{2}-9\frac{\partial W}{\partial x}\frac{\partial^{2}W}{\partial x^{2}}\frac{\partial^{3}W}{\partial x^{3}}-3{\left(\frac{\partial^{2}W}{\partial x^{2}}\right)}^{3}+\frac{45}{2}{\left(\frac{\partial W}{\partial x}\right)}^{2}{\left(\frac{\partial^{2}W}{\partial x^{2}}\right)}^{3}+\\ +\frac{45}{2}{\left(\frac{\partial W}{\partial x}\right)}^{3}\frac{\partial^{2}W}{\partial x^{2}}\frac{\partial^{3}W}{\partial x^{3}}+\frac{15}{8}{\left(\frac{\partial W}{\partial x}\right)}^{4}\left.\frac{\partial^{4}W}{\partial x^{4}}\right]-\frac{EA}{2L}\frac{\partial^{2}W}{\partial x^{2}}\left[\int_{0}^{L}{\left(\frac{\partial W}{\partial x}\right)}^{2}dx-\frac{3}{4}\right.\left.\int_{0}^{L}{\left(\frac{\partial W}{\partial x}\right)}^{4}dx\right]-\\ -\frac{2C_{0}V_{DC}^{2}}{d^{2}}\left[\frac{W}{d}+2{\left(\frac{W}{d}\right)}^{3}+3.0125{\left(\frac{W}{d}\right)}^{5}\right]-K_{1}W-K_{3}W^{3}-(K_{s}+\eta H_{x}^{2}A)\frac{\partial^{2}W}{\partial x^{2}}=F_{0}\delta (x-vt) \end{array}$$

The above equation is obtained for a simply supported microbeam such that the boundary conditions are

(21) $$W(0,t)=W(L,t)=\frac{\partial^{2}W(0,t)}{\partial x^{2}}=\frac{\partial^{2}W(L,t)}{\partial x^{2}}=0$$

To express the governing Equations (20) and (21) in nondimensional form, the following parameters are defined:

(22) $$\begin{array}{l} \bar{W}=\frac{W}{d}\begin{array}{cc} , & \bar{x}=\frac{x}{L} \end{array}\begin{array}{cc} , & \bar{t}=\frac{t}{L^{2}}\sqrt{\frac{EI}{\rho A}} \end{array}\begin{array}{cc} , & \bar{v}=vL\sqrt{\frac{\rho A}{EI}} \end{array}\begin{array}{cc} , & {\bar{F}}_{0}=\frac{F_{0}L^{5}}{dEI} \end{array},\\ \bar{\alpha }=\frac{d^{2}A}{2I}\begin{array}{cc} , & \bar{\beta }=\frac{d^{2}}{L^{2}} \end{array}\begin{array}{cc} , & {\bar{\gamma }}_{1}=\frac{2C_{0}L^{4}V_{DC}^{2}}{d^{3}EI} \end{array}\begin{array}{cc} , & {\bar{\gamma }}_{2}=\frac{L^{2}}{EI}(K_{s}+\eta AH_{z}^{2}) \end{array}\begin{array}{cc} , & {\vec{K}}_{1}=\frac{K_{1}L^{4}}{EI} \end{array}\begin{array}{cc} , & {\vec{K}}_{3}=\frac{d^{2}L^{3}K_{3}}{EI} \end{array} \end{array}$$

Omitting the bar, Equation (20) can be rewritten in nondimensional form as

(23) $$\frac{\partial^{2}W}{\partial t^{2}}+\frac{\partial^{4}W}{\partial x^{4}}-3\beta \left[\frac{1}{2}\right.\frac{\partial^{4}W}{\partial x^{4}}{\left(\frac{\partial W}{\partial x}\right)}^{2}+3\frac{\partial W}{\partial x}\frac{\partial^{2}W}{\partial x^{2}}\frac{\partial^{3}W}{\partial x^{3}}+\left.{\left(\frac{\partial^{2}W}{\partial x^{2}}\right)}^{3}\right]+\;\frac{15}{2}\beta^{2}\left[3{\left(\frac{\partial W}{\partial x}\right)}^{2}\right.{\left(\frac{\partial^{2}W}{\partial x^{2}}\right)}^{3}+3{\left(\frac{\partial W}{\partial x}\right)}^{3}\frac{\partial^{2}W}{\partial x^{2}}\frac{\partial^{3}W}{\partial x^{3}}+\frac{1}{4}{\left(\frac{\partial W}{\partial x}\right)}^{4}\frac{\partial^{4}W}{\partial x^{4}}-\alpha \frac{\partial^{2}W}{\partial x^{2}}\left[\int_{0}^{1}{\left(\frac{\partial W}{\partial x}\right)}^{2}dx-\frac{3}{4}\beta \int_{0}^{1}{\left(\frac{\partial W}{\partial x}\right)}^{4}dx\right]\;-\gamma_{1}(W+2W^{3}+3.0925W^{5})-K_{1}W-K_{3}W^{3}+\gamma_{2}\frac{\partial^{2}W}{\partial x^{2}}=F_{0}\delta (x-vt)$$

The solution of Equation (23) can be assumed to be of the following form:

(24) W(x,t)=X(x)T(t)

where, taking account of the boundary conditions (21), the eigenfunction *X*(*x*) can be expressed as

(25) $$X(x)=\sqrt{2}\sin\pi x$$

which satisfies the orthonormality condition

(26) $$\int_{0}^{1}X^{2}(x)dx=1$$

Substituting Equation (24) into (23), multiplying with *X*(*x*), integrating on the domain [0, 1], using the expression

(27) $$\int_{0}^{1}X(x)\delta (x-vz)dx=X(vt)$$

and then using the Galerkin–Bubnov procedure, Equation (23) will be written as follows:

(28) $$\ddot{T}+\omega^{2}T+aT^{3}+bT^{5}=f\sin\pi vt$$

where the dot denotes the derivative with respect to time and

(29) $$\begin{array}{l} \omega^{2}=\int_{0}^{1}\frac{d^{4}X}{dx^{4}}Xdx-(K_{1}+\gamma_{1})\int_{0}^{1}X^{2}dx\begin{array}{cc} ; & a=-\alpha \left(\int_{0}^{1}\frac{d^{2}X}{dx^{2}}Xdx\right.\left(\int_{0}^{1}{\left(\frac{dX}{dx}\right)}^{2}Xdx\right)- \end{array}\\ -\frac{3}{4}\beta \left.\int_{0}^{1}{\left(\frac{dX}{dx}\right)}^{4}Xdx\right)-3\beta \left[\frac{1}{2}\right.\int_{0}^{1}\frac{d^{4}X}{dx^{4}}{\left(\frac{dX}{dx}\right)}^{2}Xdx+3\int_{0}^{1}\frac{dX}{dx}\frac{d^{2}X}{dx^{2}}\frac{d^{3}X}{dx^{3}}Xdx+\\ +\left.\int_{0}^{1}{\left(\frac{dX}{dx}\right)}^{3}Xdx\right]-(K_{3}+2\gamma_{1})\int_{0}^{1}\frac{d^{2}X}{dx^{2}}X^{4}dx\begin{array}{cc} ; & b=\frac{3}{4}\alpha \beta \left[\int_{0}^{1}\left.\frac{d^{2}X}{dx^{2}}Xdx\right]\right.\left[\int_{0}^{1}{\left(\frac{dX}{dx}\right)}^{4}Xdx\right]- \end{array}\\ -3.0925\gamma_{1}\int_{0}^{1}X^{6}dx+\frac{15}{2}\beta^{2}\left[3\int_{0}^{1}{\left(\frac{dX}{dx}\right)}^{2}{\left(\frac{d^{2}X}{dx^{2}}\right)}^{3}Xdx\right.+\\ +3\int_{0}^{1}{\left(\frac{dX}{dx}\right)}^{3}\frac{d^{2}X}{dx^{2}}\frac{d^{3}X}{dx^{3}}Xdx+\frac{1}{4}\int_{0}^{1}{\left(\frac{dX}{dx}\right)}^{4}\left.\frac{d^{4}X}{dx^{4}}Xdx\right]\begin{array}{cc} ; & f=\sqrt{2}F_{0} \end{array} \end{array}$$

The initial conditions for Equation (28) are

(30) $$T(0)=A\begin{array}{cc} , & \dot{T}(0)=0 \end{array}$$

Equation (28) with the initial conditions (30) is a second-order nonlinear forced differential equation of the fifth order and thus is very difficult to analytically solve by an exact solution. In what follows, for Equations (28) and (29), the OHAM is applied to study the nonlinear forced vibration.

It should be mentioned that the coefficients *ω*, *a*, *b*, and *f* include the effect of the curvature, mechanical impact, electromagnetic actuation, and nonlinear elastic foundation.

## 3. The Optimal Homotopy Asymptotic Method (OHAM)

The OHAM was proposed for the first time in 2008 by Marinca and Herisanu [25,26,27,28]. To apply the OHAM, we consider the following nonlinear differential equation:

(31) $$L\left(T(t)\right)+N\left(T(t)\right)=0\begin{array}{cc} , & t\in D \end{array}\begin{array}{cc} , & B\left(T(t),\frac{dT(t)}{dt}\right)=0 \end{array}$$

where *L*[*T*(*t*)] is a linear operator, *t* denotes independent variable, *T*(*t*) is an unknown function, *N*[*T*(*t*)] is a nonlinear operator, *D* is the domain of interest, and *B* is a boundary operator.

By means of the OHAM, one first constructs a family of equations:

(32) $$(1-p)L[\bar{T}(t,p,C_{i})]=H(t,p,C_{i})\left[L(t,p,C_{i})+N(t,p,C_{i})\right]$$

where *p* ∈ [0, 1] is an embedding parameter, *H* is a nonzero auxiliary function for *p* ≠ 0 and *H*(*t*,*0*,*C_i_*) = 0, $\bar{T}$ is the unknown approximate solution, and *C_i_*, *i* = 1,2,…,n are n unknown parameters which will be determined later.

Obviously, when *p* = 0, it holds that

(33) $$L\left(T_{0}(t)\right)=0\begin{array}{cc} , & B\left(T_{0}(t),\frac{dT_{0}(t)}{dt}\right)=0 \end{array}$$

from which can be determined the initial approximation *T*_0_(*t*). If $\bar{T}\left(t\right)=T_{0}\left(t\right)+pT_{1}(t,C_{1},\ldots ,C_{n})$, then, from Equation (32), the first approximation *T*_1_(*t*, *C*_1_, *C*_2_, *…*, *C_n_*) can be determined for *p* = 1:

(34) $$L\left(T_{1}(t,C_{1},C_{2},\ldots ,C_{n})\right)=H(t,C_{1},C_{2},\ldots ,C_{n})N[T_{0}(t)]\begin{array}{cc} , & B\left(T_{1}(t),\frac{dT_{1}(t)}{dt}\right) \end{array}=0$$

where *H*(*t*, *C*_1_, *C*_2_, *…*, *C_n_*) is an arbitrary auxiliary function which can be determined such that the products *HN*[*T*_0_(*t*)] and *N*[*T*_0_(*t*)] are of the same shape. The parameters *C*_1_, *C*_2_,*….*, *C_n_* which appear in the first-order approximate solution *T*_1_ can be determined in many ways, using the least square method, the Galerkin method, the collocation method, the Ritz method, or by minimizing the square residual error as

(35) $$J(C_{1},C_{2},\ldots ,C_{n})=\int_{0}^{1}R^{2}(t,C_{1},C_{2},\ldots ,C_{n})dt$$

where *R* is given by Equation (31):

(36) $$R(t,C_{1},C_{2},\ldots ,C_{n})=L\left[\bar{T}(t)\right]+N\left[\bar{T}(t)\right]$$

The unknown parameter *C_i_* can be identified optimally from the condition

(37) $$\frac{\partial J}{\partial C_{1}}=\frac{\partial J}{\partial C_{2}}=\ldots =\frac{\partial J}{\partial C_{n}}=0$$

With these parameters known (namely, the convergence-control parameters), the approximate analytical solution *T*(*t*) is well determined.

It should be emphasized that the auxiliary function and the number n of the convergence-control parameters are not unique.

The OHAM is a very efficient procedure since any nonlinear differential equation is reduced to only two linear differential equations, and, on the other hand, the convergence-control parameters are optimally determined.

## 4. Application of Optimal Homotopy Asymptotic Method to Study the Nonlinear Vibration of the Microbeam

In what follows, we apply the OHAM to obtain an approximate solution for Equations (28) and (30). Making the transformations

(38) $$\tau =\Omega t\begin{array}{cc} , & T(t)=A\theta (\tau ) \end{array}$$

where Ω is the unknown natural frequency of the beam, Equation (28) becomes

(39) $$\theta^{\prime \prime }+\frac{\omega^{2}}{\Omega^{2}}\theta +\frac{aA^{2}}{\Omega^{2}}\theta^{3}+\frac{bA^{4}}{\Omega^{2}}\theta^{5}-\frac{f}{A\Omega^{2}}\sin\pi \frac{v\tau }{\Omega }=0$$

and corresponding initial conditions are

(40) $$\begin{array}{cc} & \theta (0)=1 \end{array}\begin{array}{cc} , & \theta^{\prime }(0)=0 \end{array}$$

where the prime denotes the derivative with respect to *τ*.

The linear and nonlinear operators are, respectively,

(41) $$L[\theta (\tau )]=\theta^{\prime \prime }+\theta \begin{array}{cc} ; & N[\theta (\tau )]=\left(\frac{\omega^{2}}{\Omega^{2}}-1\right)\theta +\frac{aA^{2}}{\Omega^{2}}\theta^{3}+\frac{bA^{4}}{\Omega^{2}}\theta^{5}-\frac{f}{A\Omega^{2}}\sin\pi \frac{v\tau }{\Omega } \end{array}$$

The initial approximation is determined from Equation (33), which is rewritten as

(42) $${\theta^{\prime \prime }}_{0}+\theta_{0}=0\begin{array}{cc} , & \theta_{0}(0)=1 \end{array}\begin{array}{cc} , & {\theta^{\prime }}_{0}(0)=0 \end{array}$$

whose solution is

(43) $$\theta_{0}(\tau )=\cos\tau$$

The first approximation *θ*_1_(*τ*) is obtained from Equation (34) as

(44) $${\theta^{\prime \prime }}_{1}+\theta_{1}=H(\tau ,C_{1},C_{2},\ldots ,C_{n})N[\theta_{0}(\tau )]\begin{array}{cc} , & \theta_{1}(0)= \end{array}{\theta^{\prime }}_{1}(0)=0$$

where *N*[*θ*_0_(*τ*)] is determined by inserting Equation (43) into the nonlinear operator (41):

(45) $$N[\theta_{0}(\tau )]=A_{1}\cos\tau +A_{3}\cos3\tau +A_{5}\cos5\tau -\frac{f}{A\Omega^{2}}\sin\frac{\pi v\tau }{\Omega }$$

The coefficients from the above equations are

(46) $$A_{1}=\frac{\omega^{2}}{\Omega^{2}}-1+\frac{3aA^{2}}{4\Omega^{2}}+\frac{5bA^{4}}{8\Omega^{2}}\begin{array}{cc} , & A_{3}=\frac{aA^{2}}{4\Omega^{2}}+ \end{array}\frac{5bA^{4}}{16\Omega^{2}}\begin{array}{cc} , & A_{5}=\frac{bA^{4}}{16\Omega^{2}} \end{array}$$

The auxiliary function *H* from Equation (44) is chosen such that the product between *HN*[*θ*_0_(*τ*)] and *N*[*θ*_0_(*τ*)] is of the same form. For example, we can choose these auxiliary functions in the following expressions:

(47) $$H_{1}(t,C_{1},C_{2},C_{3},C_{4})=C_{1}+2C_{2}\cos2\tau +2C_{3}\cos4\tau +2C_{4}\cos6\tau$$

(48) $$H_{2}(t,C_{1},C_{2},C_{3})=C_{1}+2C_{2}\cos2\tau +2C_{3}\cos4\tau$$

(49) $$H_{3}(t,C_{1},C_{2},C_{3},C_{4},C_{5})=C_{1}+2C_{2}\cos2\tau +2C_{3}\cos4\tau +2C_{4}\cos6\tau +2C_{5}\cos8\tau$$

(50) $$H_{4}(t,C_{1},C_{2})=C_{1}+2C_{2}\cos4\tau$$

Using only expression (47), Equation (44) can be rewritten after some manipulations as follows:

(51) $$\begin{array}{l} {\theta^{\prime \prime }}_{1}+\theta_{1}=[A_{1}(C_{1}+C_{2})+A_{3}(C_{2}+C_{3})+A_{5}(C_{3}+C_{4})]\cos\tau +[A_{3}(C_{1}+C_{4})+\\ +A_{1}(C_{2}+C_{3})+A_{5}C_{2}]\cos3\tau +[A_{5}C_{1}+A_{3}C_{2}+A_{1}(C_{3}+C_{4})]\cos5\tau +(A_{5}C_{2}+\\ +A_{3}C_{3}+A_{1}C_{4})\cos7\tau +(A_{5}C_{3}+A_{3}C_{4})\cos9\tau +A_{5}C_{4}\cos11\tau \begin{array}{cc} , & \theta_{1}(0)= \end{array}{\theta^{\prime }}_{1}(0)=0 \end{array}$$

No secular term in Equation (51) requires that the coefficient of cosτ be zero. From this condition, one can determine the natural frequency:

(52) $$\Omega^{2}=\omega^{2}+\frac{3aA^{2}}{4}+\frac{5bA^{4}}{8}+\left(\frac{aA^{2}}{4}+\frac{5bA^{4}}{16}\right)\frac{C_{2}+C_{3}}{C_{1}+C_{2}}+\frac{bA^{4}}{16}\frac{C_{3}+C_{4}}{C_{1}+C_{2}}$$

The solution of linear Equation (51) is

(53) $$\begin{array}{l} \theta_{1}(\tau )=\frac{A_{3}(C_{1}+C_{4})+A_{1}(C_{2}+C_{3})+A_{5}C_{2}}{8}(\cos\tau -\cos3\tau )+\\ +\frac{A_{5}C_{1}+A_{3}C_{2}+A_{1}(C_{3}+C_{4})}{24}(\cos\tau -\cos5\tau )+\frac{A_{5}C_{2}+A_{3}C_{3}+A_{1}C_{4}}{48}(\cos\tau -\\ \cos7\tau )+\frac{A_{5}C_{3}+A_{3}C_{4}}{80}(\cos\tau -\cos9\tau )+\frac{A_{5}C_{4}}{120}(\cos\tau -\cos11\tau ) \end{array}$$

The approximate solution $\bar{T}$ is obtained from Equations (38), (41) and (55):

(54) $$\begin{array}{l} \bar{T}(t)=A\cos\Omega t+\frac{A[A_{3}(C_{1}+C_{4})+A_{1}(C_{2}+C_{3})+A_{5}C_{2}]}{8}(\cos\Omega t-\cos3\Omega t)+\\ +\frac{A[A_{5}C_{1}+A_{3}C_{2}+A_{1}(C_{3}+C_{4})]}{24}(\cos\Omega t-\cos5\Omega t)+\\ \frac{A[A_{5}C_{2}+A_{3}C_{3}+A_{1}C_{4}]}{48}(\cos\Omega t-\cos7\Omega t)+\frac{A[A_{5}C_{3}+A_{3}C_{4}]}{80}(\cos\Omega t-\cos9\Omega t)+\\ +\frac{AA_{5}C_{4}}{120}(\cos\Omega t-\cos11\Omega t) \end{array}$$

where Ω is given by Equation (32).

The OHAM can be applied to any nonlinear differential equations without a supplementary hypothesis, such as, for example, the existence of a small parameter in the differential equation or in the boundary/initial conditions.

## 5. Numerical Results

The efficiency of the OHAM can be proved through numerical investigations for the following case considering *α* = 0.007, *β* = 0.001, *γ*_1_ = 0.3, *γ*_2_ = 0.5, *k*_1_ = 1, and *k*_3_ = 0.4. It follows that *ω* = 1.1, *a* = 0.023, *b* = 0.03, *f* = 0.03, and *v* = 0.95.

The optimal values of the convergence-control parameters are obtained by minimizing the residual error: *C*_1_ = 0.430116872481, *C*_2_ = 0.000287558193, *C*_3_ = 0.006874588249, *C*_4_ = 0.049982573619, and Ω = 1.116.

**Figure 2:**
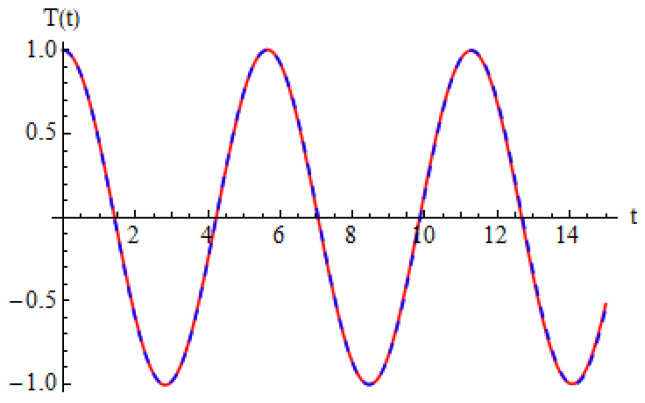
Comparison between analytical OHAM solution and numerical integration results: **____** = numerical, **_ _ _** = analytical.

To validate the obtained analytical solution, the analytical results are compared with numerical integration results obtained by means of a fourth-order Runge–Kutta approach. The explicit analytical solution benefits from the optimal values identified for the convergence-control parameters and is plotted in Figure 2, demonstrating the high accuracy of the employed method. In Figure 2, we present the approximate solution (54) in comparison with the numerical integration results. It can be observed that the error between the approximate analytical solution obtained by the OHAM and the solution obtained by numerical integration is very small, these solutions being in an excellent agreement. In this way, the results obtained by the OHAM are validated through this comparison, and an excellent accuracy of the analytical solution is proved.

## 6. Analysis of the Stability of the Steady-State Motion near the Primary Resonance

The procedure applied in this section proposes to distinguish between two time-scales by associating a separate independent variable with each one and using the Homotopy Perturbation Method [29]. We reconsider the primary resonance for Equation (28):

(55) $$\omega =\bar{\omega }+\bar{\lambda }p\begin{array}{cc} , & \bar{\omega }=\pi v \end{array}$$

with *λ* being a detuning parameter and *p* ∈ [0, 1] the embedding parameter. As in Section 3,

(56) $$\xi =\omega t\begin{array}{cc} , & \eta =pt \end{array}$$

In accordance with the Homotopy Perturbation Method, for *p* = 0, any nonlinear differential equation reduces to a linear differential equation, and, for *p* = 1, one obtains the original equation, such that the original Equation (28) is rewritten as

(57) $$\ddot{T}+\omega^{2}T+p(aT^{3}+bT^{5}-f\sin\bar{\omega }t)=0$$

The variable *T* can be written as a power series of *p*:

(58) $$T=S_{0}+pS_{1}+p^{2}S_{2}+\ldots$$

where *S_i_* are the solutions of the i-th order homotopy equations.

To substitute Equation (56) into (57), we need the following expressions:

(59) $$\frac{dT}{dt}=\frac{\partial T}{\partial \xi }\frac{\partial \xi }{\partial t}+\frac{\partial T}{\partial \eta }\frac{\partial \eta }{\partial t}=\omega \frac{\partial T}{\partial \xi }+p\frac{\partial T}{\partial \eta }$$

(60) $$\ddot{T}=\frac{d^{2}T}{dt^{2}}=\omega^{2}\frac{d^{2}T}{d\xi^{2}}+2\omega p\frac{d^{2}T}{d\xi \partial \eta }+p^{2}\frac{d^{2}T}{d\eta^{2}}$$

Substituting Equations (57) and (58) into Equation (60), one can obtain

(61) $$\ddot{T}={\bar{\omega }}^{2}\frac{\partial^{2}S_{0}}{\partial \xi^{2}}+p\left[{\bar{\omega }}^{2}\frac{\partial^{2}S_{1}}{\partial \xi^{2}}+2\bar{\omega }\bar{\lambda }\frac{\partial^{2}S_{0}}{\partial \xi^{2}}+2\bar{\omega }\frac{\partial^{2}S_{0}}{\partial \xi \partial \eta }\right]+\ldots$$

From Equations (57), (58) and (61), the homotopy equations of order *p^i^*, *i* = 0,1 are, respectively,

(62) $$p^{0}\begin{array}{cc} : & {\bar{\omega }}^{2}\left(\frac{\partial^{2}S_{0}}{\partial \xi^{2}}+S_{0}\right) \end{array}=0$$

(63) $$p^{1}\begin{array}{cc} : & {\bar{\omega }}^{2}\left(\frac{\partial^{2}S_{1}}{\partial \xi^{2}}+S_{1}\right) \end{array}+2\bar{\lambda }\bar{\omega }\frac{\partial^{2}S_{0}}{\partial \xi^{2}}+2\bar{\omega }\frac{d^{2}T_{0}}{d\xi \partial \eta }+2\bar{\lambda }\bar{\omega }S_{0}+aS_{0}^{3}+bS_{0}^{5}-f\sin\xi =0$$

The solution of the linear Equation (62) with partial derivatives is

(64) $$S_{0}(\xi ,\eta )=A(\eta )\cos\xi +B(\eta )\sin\xi$$

where *A* and *B* are functions depending on *η*.

Now, substituting Equation (64) into Equation (63) and avoiding the secular terms yields

(65) $$2\bar{\omega }\frac{dB}{d\eta }+\frac{3aA}{4}(A^{2}+B^{2})+\frac{5bA}{8}{\left(A^{2}+B^{2}\right)}^{2}=0$$

(66) $$-2\bar{\omega }\frac{dA}{d\eta }+\frac{3aB}{4}(A^{2}+B^{2})+\frac{5bB}{8}{\left(A^{2}+B^{2}\right)}^{2}-f=0$$

The equilibrium points correspond to the periodic solution of Equation (28). To determine them, set the following:

(67) $$\frac{dA}{d\eta }=\frac{dB}{d\eta }=0$$

In this way, the equilibrium points can be obtained from the algebraic equations

(68) $$A_{e}\left[6a+5b(A_{e}^{2}+B_{e}^{2})=0\right]$$

(69) $$\left[6a+5b(A_{e}^{2}+B_{e}^{2})\right]B_{e}(A_{e}^{2}+B_{e}^{2})=8f$$

It is possible only in the case where

(70) $$A_{e}=0\begin{array}{cc} , & 5bB_{e}^{5}+6aB_{e}^{3}-8f=0 \end{array}$$

**Figure 3:**
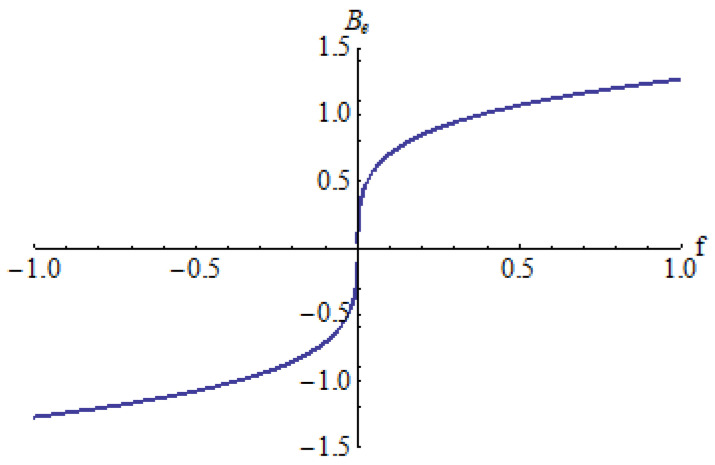
Equilibrium point *B_e_* for *a* = 0.25, *b* = 0.3.

**Figure 4:**
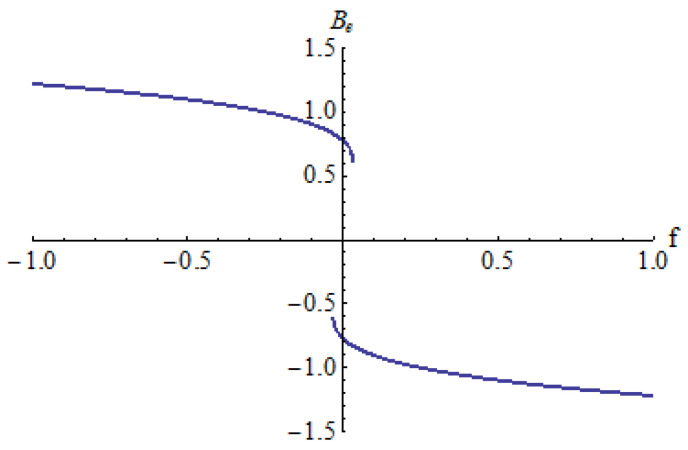
Equilibrium point *B_e_* for *a* = 0.5, *b* = 1.

**Figure 5:**
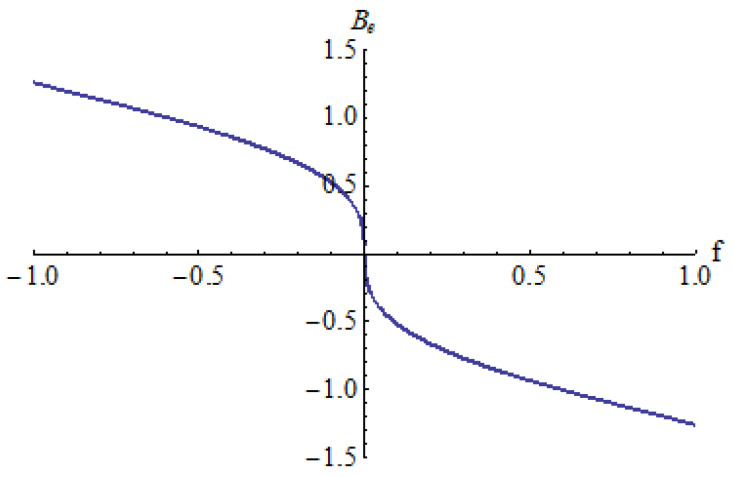
Equilibrium point *B_e_* for *a* = −1, *b* = 0.25.

In Figure 3, Figure 4 and Figure 5 is depicted the equilibrium point *B_e_* in respect to the parameter *f* for different values of the parameters *a* and *b*: *a* = 0.25, *b* = 0.3 (Figure 3); *a* = 0.5, *b* = 1 (Figure 4); *a* = −1, *b* = 0.25 (Figure 5).

It can be seen that there is a single equilibrium point B_e_ in every case, except in situations where there are two equilibrium points *B_e_* in a small neighborhood of *f* = 0 for *a* = 0.5 and *b* = 1 (Figure 4).

The stability of the steady-state motion is determined by the eigenvalues of the Jacobian matrix obtained from Equations (66) and (67) (the Routh–Hurwitz criteria):

(71) $$[J]=\left[\begin{array}{cc} a_{11} & a_{12}\\ a_{21} & a_{22} \end{array}\right]$$

where

(72) $$a_{11}={\left.\frac{\partial A\prime }{\partial A}\right|}_{\left(A_{e},B_{e}\right)}\begin{array}{cc} ; & a_{12}= \end{array}{\left.\frac{\partial A\prime }{\partial B}\right|}_{\left(A_{e},B_{e}\right)}\begin{array}{cc} ; & a_{21}={\left.\frac{\partial B\prime }{\partial A}\right|}_{\left(A_{e},B_{e}\right)} \end{array}\begin{array}{cc} ; & a_{22}={\left.\frac{\partial B\prime }{\partial A}\right|}_{\left(A_{e},B_{e}\right)} \end{array}\begin{array}{cc} ; & A\prime =\frac{\partial A}{\partial \eta } \end{array},B\prime =\frac{\partial B}{\partial \eta }$$

After some manipulations, from Equations (66), (67) and (69), the coefficients *a_ij_* have the form

(73) $$a_{11}=a_{22}=0\begin{array}{cc} , & a_{12}=\frac{(18a+25bB_{e}^{2})B_{e}^{2}}{16\bar{\omega }} \end{array}\begin{array}{cc} , & a_{21}=-\frac{(6a+5bB_{e}^{2})B_{e}^{2}}{16\bar{\omega }} \end{array}$$

The eigenvalues of the Jacobian matrix are obtained from the characteristic equation

(74) $$\det\left(\left[J\right]-\lambda \left[I\right]\right)=0$$

where *I* is the unity matrix of the second order, and *λ* is the eigenvalue of the Jacobian matrix. The characteristic equation can be rewritten as

(75) $$\lambda^{2}+\frac{(18a+25bB_{e}^{2})(6a+5bB_{e}^{2})B_{e}^{4}}{256{\bar{\omega }}^{2}}=0$$

We have the following possible cases:

Case 6.1 If *ab* ≥ 0 and *a*^2^ + *b*^2^ ≠ 0, then the eigenvalues are purely imaginary with opposite signs of their imaginary parts. This is a center, such that there is no net motion towards or away from the equilibrium point.

Case 6.2 If *ab* < 0 and $B_{e}^{2}=-\frac{18a}{25b}$ or $B_{e}^{2}=-\frac{6a}{5b}$, then *λ* _1_= *λ* _2_ = 0. The situation is similar to the above.

Case 6.3 If *ab* < 0 and $-\frac{18a}{25b}<B_{e}^{2}<-\frac{6a}{5b}$, then *λ*_1_ and *λ*_2_ are real, and *λ*_1_ = −*λ*_2_. This case corresponds to a saddle point.

Case 6.4 If *ab* < 0 and $B_{e}^{2}<-\frac{18a}{25b}$ or $B_{e}^{2}>-\frac{6a}{5b}$, then the eigenvalues are purely imaginary, as in the situation of case 6.1.

## 7. Results and Discussion

**Figure 6:**
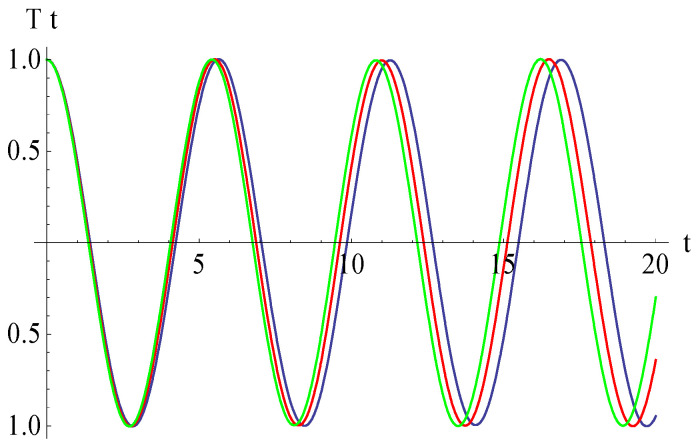
Effects of the curvature parameter *β* on the vertical displacement: *β* = 0.01 (blue), *β* = 0.02 (red), *β* = 0.03 (green).

**Figure 7:**
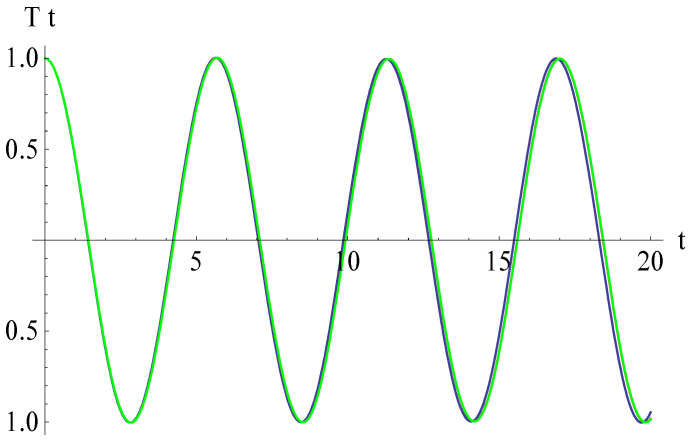
Effects of the nonlinear Winkler–Pasternak parameter *K*_3_ on the vertical displacement: *K*_3_ = 0.4 (blue), *K*_3_ = 0.5 (red), *K*_3_ = 0.6 (green).

**Figure 8:**
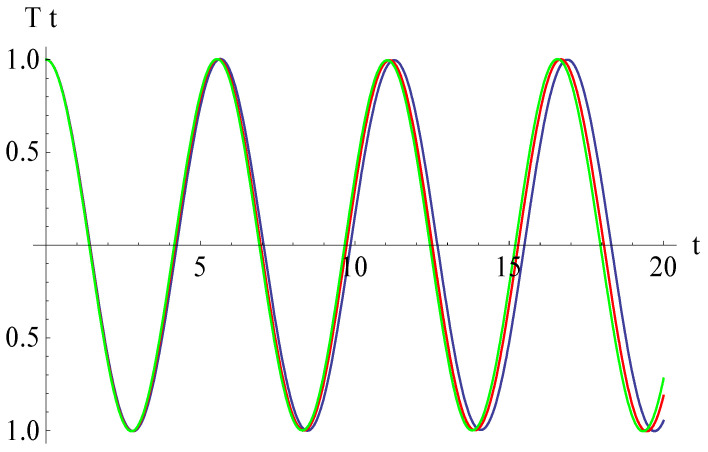
Effects of the gap parameter *α* on the vertical displacement: *α* = 0.07 (blue), *α* = 0.08 (red), *α* = 0.09 (green).

**Figure 9:**
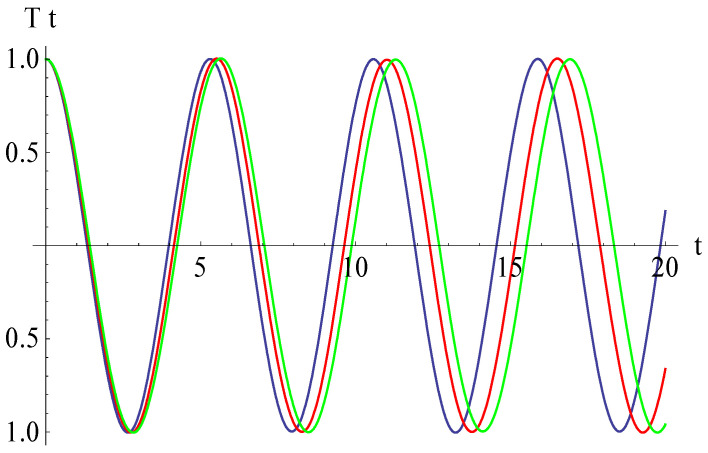
Effects of the electromagnetic parameter *γ*_1_ on the vertical displacement: *γ*_1_ = 0.3 (blue), *γ*_1_ = 0.4 (red), *γ*_1_ = 0.5 (green).

In Figure 6, Figure 7, Figure 8 and Figure 9, the effects of different parameters on the vertical displacements of the Euler–Bernoulli beam are shown.

From Figure 6, we deduce that, if the curvature parameter *β* increases, then the abscissa of the intersection point of the vertical displacement and the horizontal axis decreases. Different values of *β* are taken as 0.01, 0.02, and 0.03. The effect of the nonlinear Winkler–Pasternak parameter *K*_3_ on the vertical displacement is shown in Figure 7. This figure shows that, if the parameter increases from *K*_3_ = 0.4 to *K*_3_ = 0.6, then the abscissa of the intersection point of the vertical displacement and the horizontal axis increases. The effect of the gap parameter α is illustrated in Figure 8; the abscissa of the intersection point of the vertical displacement and the horizontal axis decreases with the growth of the gap parameter. From Figure 9, it follows that, if the electromagnetic parameter *γ*_1_ increases, then the above-mentioned abscissa increases even more substantially for the time *t* > 14.

It can be observed that the longitudinal inertia term *u*(*x,t*) can be neglected only if *E/ρ* satisfies the condition (83).

## 8. Conclusions

According to the present results, the oscillatory behavior of a simply supported flexible uniform microbeam is studied. The Euler–Bernoulli microbeam is subjected to a mechanical impact using the Dirac delta function, an electromagnetic actuation, a nonlinear Winkler–Pasternak foundation, and a longitudinal magnetic field. In the governing differential equations, the curvature of the beam, the second-order approximation of the deflected beam, and the boundary conditions are taken into consideration. The OHAM is applied to solve the complex nonlinear differential equation, introducing so-called auxiliary functions and some convergence-control parameters without supplementary hypotheses. These parameters are optimally determined by rigorous mathematical procedures. The proper procedure leads to a very accurate solution using only the first iteration. It should be emphasized that the nonlinear dynamical system is reduced to only two linear differential equations.

The main aspects of novelty presented in this work, which are not present in previous related research, are summarized as follows:

(a) The combination of the effects of the curvatures, mechanical impact, electromagnetic actuation, nonlinear elastic Winkler–Pasternak foundation, and longitudinal magnetic field. This combination is ensured by the new dynamical model which is considered in the study.
(b) The Optimal Homotopy Asymptotic Method is used to solve a very complex differential equation by means of some auxiliary functions, providing novel solutions.
(c) The values of the convergence-control parameters are optimally determined, ensuring a high degree of accuracy.
(d) It is proved that the longitudinal inertia term can be neglected, but only if *E/ρ* satisfies the condition (83).
(e) Local stability for the primary resonance is studied by means of the Homotopy Perturbation Method.

The effect of some physical parameters is highlighted. The local stability depends on the nature of the solutions to the characteristic equation, and different cases are discussed. The local stability near the primary resonance is achieved by using the Homotopy Perturbation Method, the variable expansion method, equilibrium points, and the Jacobian matrix. The effects of different parameters on the vertical displacements of the beam are considered.

## Data Availability

All data are presented within the paper.
